# Metal nanozyme sensors with different modes: biomedical applications and research advances

**DOI:** 10.3389/fbioe.2026.1795569

**Published:** 2026-04-22

**Authors:** Banghui Wang, Tianyi Zhang, Meiling Zhao, Weiwei Song, Maojin Tian

**Affiliations:** 1 Department of Cardiology, The Affiliated Hospital of Qingdao University, Qingdao, China; 2 Department of Critical Care Medicine, Shandong Provincial Hospital affiliated to Shandong First Medical University, Jinan, China; 3 Department of Critical Care Medicine, Zibo Central Hospital, Zibo, China; 4 College of Life Science, Institute of Biomedical Engineering, Qingdao University, Qingdao, China

**Keywords:** biomedical applications, dual-mode sensing, metal nanozymes, multi-mode sensing, single-mode sensing

## Abstract

In recent years, with the continuous advancement of nanotechnology, nanozymes have demonstrated tremendous potential in biomedical applications. By mimicking the catalytic activities of biological enzymes such as oxidases, peroxidases, and superoxide dismutases, nanozymes can be employed to detect various analytes, enabling their application in sensors. In the research of nanozymes, owing to their unique electronic structures and abundant surface active sites, endow metal nanozymes with both tunable high activity and strong stability. They can not only efficiently mimic the catalytic functions of various natural enzymes such as peroxidases, oxidases, and superoxide dismutases, but some types also exhibit high catalytic efficiency, even surpassing the natural enzymes themselves. This ability to mimic enzymatic activity endows nanozymes with distinctive properties. Signal conversion modes for metal nanozymes encompass multiple forms, including colorimetric, electrochemical, Surface-Enhanced Raman Scattering, and fluorescent detection. Herein, this review categorizes metal nanozyme sensors with different sensing modes into three major types: single-mode, dual-mode, and multi-mode. It summarizes the application potential of various sensor modes in scenarios such as biomarker detection, pathogen screening, and disease diagnosis, while analyzing current challenges including catalytic specificity and mode-to-mode synergy. Furthermore, this review explores the core challenges confronting metal nanozyme sensors and outlines future development directions, providing insights for the research and development of novel sensing technologies and their biomedical applications.

## Graphical Abstract

**Figure:**
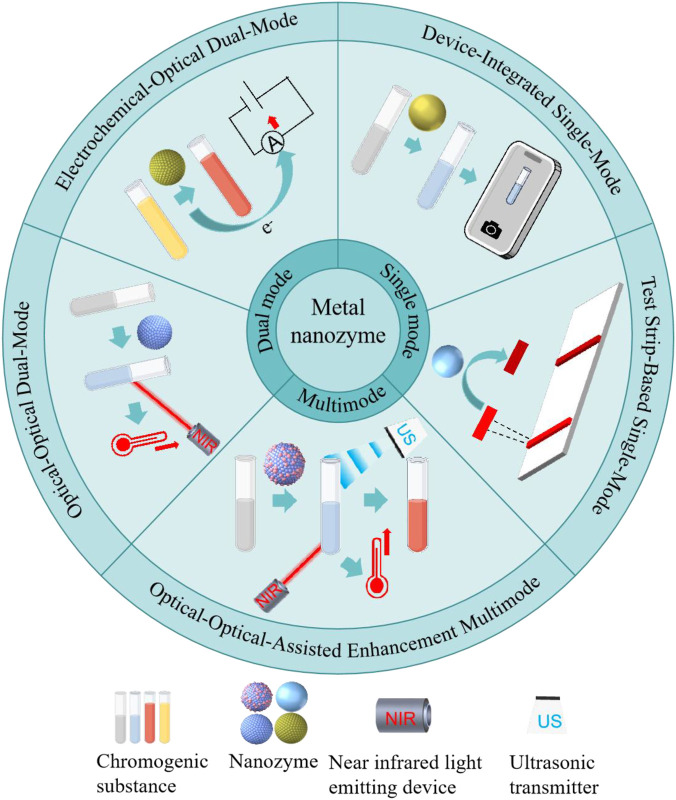
Created with biogdp.com: GDP2025IJW3J4, by figdraw: OAUIR0b4b3.

## 1 Introduction

As the core carrier of biomedical detection technology, the performance improvement of sensors is highly dependent on breakthroughs in recognition components and signal conversion mechanisms (Yu et al., 2024a). The ultimate goal is to achieve rapid, high-sensitivity, and specific identification of target analytes and reliable signals in complex biomatrices (Yu et al., 2024b). Although traditional biosensors mostly employ natural enzymes as biorecognition and catalytic units and exhibit excellent substrate specificity, they are plagued by several inherent limitations, including high preparation costs, poor environmental stability, difficult long-term storage, and challenges in large-scale production. These drawbacks significantly restrict their practical applications in long-term and dynamic detection scenarios such as clinical diagnosis and pathological monitoring (Liu S. et al., 2024; Huang et al., 2019). In this context, the discovery and rise of nanozymes provide revolutionary ideas and key material support to solve this dilemma (Jiang D. et al., 2019). This type of nanomaterials with enzyme-like catalytic activity not only inherits the catalytic function of natural enzymes, but also has the advantages of strong chemical stability, simple preparation process, and high modifiability, and has become a research hotspot in the field of biosensors (Wu et al., 2019; Wei and Wang, 2013; Wang K. et al., 2025).

Traditional biosensors mostly employ natural enzymes as biorecognition and catalytic units and thus exhibit excellent substrate specificity. However, they are limited by high preparation costs, poor environmental stability, difficult long-term storage and challenges in large-scale production, which significantly restricts their applications in long-term and dynamic detection scenarios like clinical diagnosis and pathological monitoring (Somerville et al., 2023; Shang et al., 2023; Liu L. et al., 2021). At present, the needs of biomedicine are changing rapidly, and the testing needs are shifting from qualitative analysis of a single marker to quantitative and synchronous monitoring of multiple indicators to obtain more comprehensive information. This trend places higher demands on the accuracy, anti-interference ability, and information dimension of sensing technology (Akdogan et al., 2022; Wu et al., 2018; Wu et al., 2021). Single-mode nanozyme sensors represent the most technologically mature and rapidly commercialized sensing systems currently available on the market. Replacing traditional colloidal gold with nanozymes can significantly enhance the detection sensitivity of sensors. Sensors with a single detection mode can no longer meet the needs of accurate analysis of complex markers, as they often struggle to ensure the reliability and accuracy of complex biological samples (Zhang et al., 2022). To address these challenges, the development of multimodal metal nanozyme sensing platforms has become a key technical direction. By intelligently integrating multiple signal conversion mechanisms (*e.g.*, electrochemical, optical), these platforms leverage the synergistic complementarity between different modes to effectively break through the limitations of any single method. This not only improves detection accuracy and provides built-in self-correction capabilities but also enables cross-validation, making them uniquely suitable for diverse and demanding biomedical detection scenarios (Wang et al., 2021; Huang et al., 2021; Liu et al., 2023). Although multimodal sensors offer advantages such as interference resistance and greater measurement accuracy, they remain in the research and development or clinical validation stages and have not yet been widely commercialized.

However, the existing reviews focus on a single detection mode and lack a systematic review of the mechanism of action of metal nanozymes in multiple modes, making it difficult to form a global cognition (Falahati et al., 2022; Wang and Xianyu, 2022; Wang B. et al., 2025). Although these studies have made outstanding contributions to this field, there is currently a lack of systematic reviews of different metal nanozyme sensing platform models, and there is a lack of comparative analysis of the advantages and disadvantages of different sensing platform models. This review aims to systematically review and evaluate the latest breakthrough applications and research progress of metal nanozyme sensing platforms in the field of biomedicine. By intelligently integrating multiple signal conversion mechanisms (*e.g.*, electrochemical, optical), these platforms leverage the synergistic complementarity between different modes to effectively break through the limitations of any single detection method. They thus achieve improved detection accuracy, built-in self-correction capabilities and cross-validation functionality, rendering them uniquely suitable for diverse and demanding biomedical detection scenarios. Single-mode sensing includes test strip-based single-mode sensing, where we focus on specific applications in lateral flow immunochromatography, and device-integrated single-mode sensing, where we explore the construction of highly sensitive sensing platforms based on devices such as smartphones. Dual-mode sensing includes electrochemical-optical dual-mode sensing and optical-optical dual-mode sensing, which elucidate the adaptation mechanism of different metal nanozymes. Multi-mode sensing emphasizes how to achieve multi-functional and high-precision detection and application by integrating multiple enzyme activities and signaling mechanisms. This review aims to establish a clear and profound theoretical understanding for readers through systematic integration, in-depth comparison and future prospects of cutting-edge research. Its mission is not only to summarize the results, but also to gain insight into future trends and generate innovative inspiration, so as to lay the theoretical cornerstone and inject design wisdom into the next-generation of intelligent, accurate, efficient, and clinically promising multimodal metal nanozyme sensing platforms (Yu et al., 2024b; Chen et al., 2024; Wen et al., 2024).

## 2 Single-mode metal nanozyme sensors

Single-mode metal nanozyme sensors represent a fundamental and critical research branch of metal nanozyme sensing technology due to the specificity of their signal output mechanism and the simplicity of their detection systems. These sensors leverage the catalytic activity of metal nanozymes to generate quantifiable, singular signals through substrate catalysis, enabling precise identification and quantitative detection of disease biomarkers. This section focuses on recent advancements in device-integrated metal nanozyme sensors and test strip-based metal nanozyme sensors within the single-mode paradigm. A comparative analysis table (Table 1) highlights the similarities and differences among various single-mode metal nanozyme sensors, aiming to provide broad insights for future research directions.

**TABLE 1:** Comparison of single-mode metal nanozyme sensors.

Category	Nanozyme	Structure	Technique	Target	Application	Ref.
Device-Integrated Single-Mode Sensing	CuCo_2_S_4_	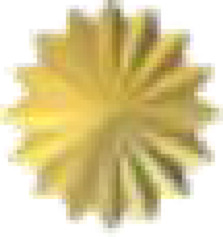	1) Stimulus-responsive hydrogel kit 2) Smartphone imaging 3) ImageJ analysis	Uric acid	Rapid on-site detection of uric acid	Yuan et al. (2022)
	MnB_2_	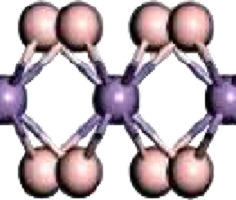	1) Microfluidic paper-based analytical device 2) Analyze images using an in-smartphone app (ctDNA Detection)	ctDNA	Point-of-care detection of lung cancer with liquid biopsy	Shi et al. (2024a)
	CuCo_2_O_4_	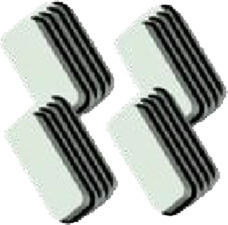	1) Paper-based sensor arrays 2) Smartphone imaging 3) ImageJ analysis	Alkaloid	Rapid identification and differentiation of alkaloids	Liu et al. (2024b)
	FeCoZn-TAC/SNC	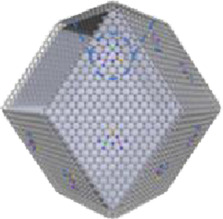	Three-atomic nanozyme design	Ascorbic acid	Sensitive detection of ascorbic acid	Wu et al. (2022)
	Mn/Fe COP	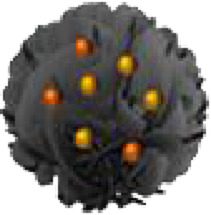	1) Smartphone-assisted detection 2) Temperature is regulated to synthesize nanozymes	1) Ascorbic acid 2) α-Glu 3) α-Glu inhibitors	1) Detection of α-Glu activity in human serum 2) Assists in diabetes drug development	Jiang et al. (2025)
	Ti_3_C_2_TxNR@AuNPs	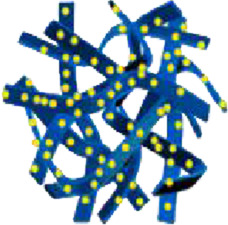	1) Self-reduction synthesis method 2) Smartphone colorimetric analysis	1) Mercury ions 2) Cysteine	Analysis of the biomarker cysteine in human serum	Zhou et al. (2023)
​	PtPd	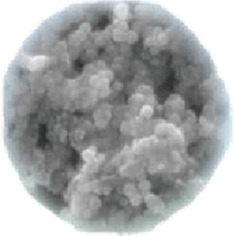	1) Nanozymes are prepared by one-pot method 2) Differentiated induction of three-channel colorimetric sensor array	Antioxidant	Simultaneous identification of multiple antioxidants in serum samples	Liu et al. (2026)
	H–MnO_2_	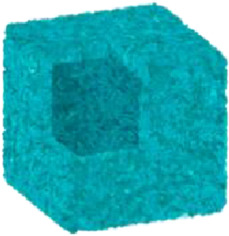	1) Self-template sacrifice method 2) Smartphone colorimetric analysis	1) Aspartate aminotransferase 2) Alanine aminotransferase 3) Alkaline phosphatase	1) Liver function test 2) Real-time health monitoring	Li et al. (2023a)
	Ni_0.75_Co_0.25_Se	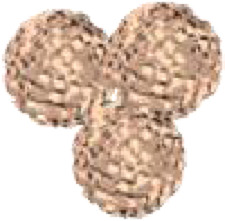	1) Hydrothermal synthesis and selenization method 2) 3D printing dark room 3) Smartphone assistance	Hydrogen peroxide	It can be used as a basic sensor to further develop a platform for the detection of biomarkers	Lian et al. (2024)
	L-Arg CeO_2_	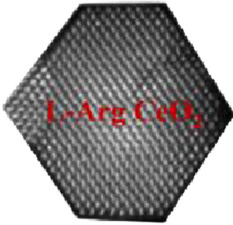	Hydrothermal synthesis method	1) Fluoride ions 2) Zearalenone 3) Hydrogen peroxide	Anti-interference sensing	Xiong et al. (2022)
Test Strip-Based Single-Mode Sensing	Au@Ag-Pt	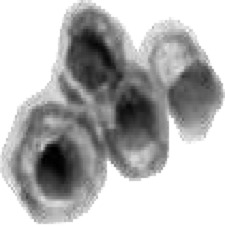	Three-step hydrothermal synthesis method	CRP	Point-of-care detection of inflammatory markers	Panferov et al. (2021)
	FeWO_4_	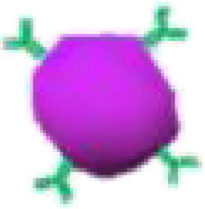	One-step hydrothermal method	CRP	Point-of-care detection of inflammatory markers	​
	FeMC6*a	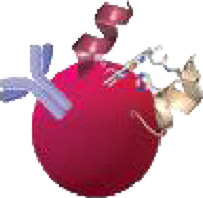	Gold nanoparticle functionalization	Human immunoglobulin G	Point-of-care immunoassay	Renzi et al. (2023)
	Au@CeO_2_	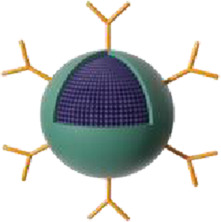	One-pot redox reaction	Cardiac fatty acid-binding proteins	Early diagnosis and monitoring of acute myocardial infarction	Wang H. et al. (2024)

### 2.1 Device-integrated single-mode sensing

Device-Integrated Single-Mode Sensing technology simulates the enzyme activity of biological enzymes through metal nanozymes, cooperates with the device for sensing with its excellent catalytic effect, and constructs a sensing system with precise synergistic effect (Ya et al., 2024). With high sensitivity, specificity and detection accuracy, this technology is widely used in biomedical real-time detection scenarios, such as circulating tumor DNA (ctDNA) liquid biopsy and blood glucose monitoring of diabetes patients (Jiang et al., 2025). The core design concept is to generate colorimetric signals through the specific catalysis of metal nanozymes, simplify the signal reading process of devices, and ultimately achieve rapid and accurate quantitative detection of target substances (Cai et al., 2022; Guo et al., 2017; Yao et al., 2021).

**FIGURE 1:**
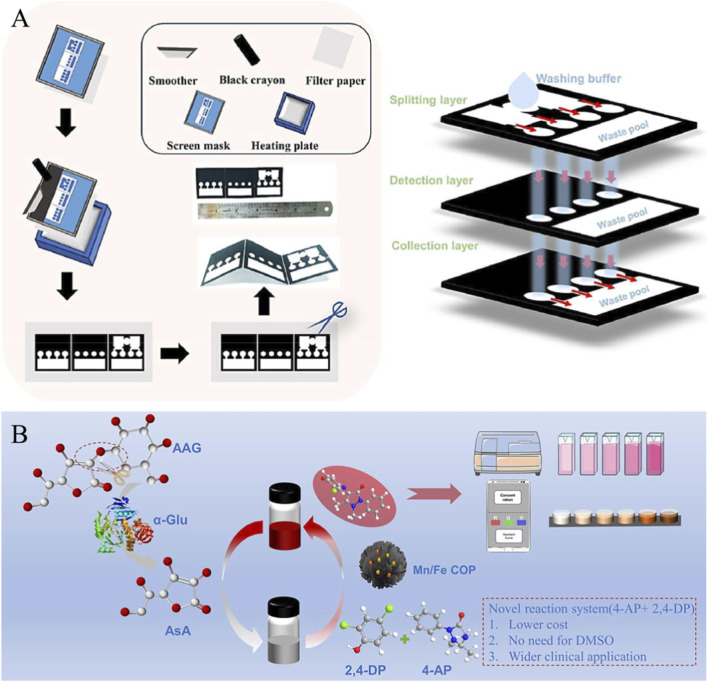
**(A)** The microfluidic paper-based analytical device (μPAD) introduced in this paper adopts a three-layer structure: flow splitting, detection, and collection layer. **(B)** Quantification of α-Glu is achieved through dual-mode detection using spectrophotometric colorimetry and smartphone RGB recognition. Reprinted with permission from Jiang et al. (2025) and Ya et al. (2024).

To address the need for rapid detection of ctDNA in liquid biopsies for non-small cell lung cancer, Yang et al. developed a smartphone-integrated microfluidic system with MnB_2_ nanozymes as the catalytic core (Ya et al., 2024). The sensing mechanism employs a microfluidic paper-based analytical device (μPAD) and a sandwich-type nucleic acid hybridization mode: the μPAD features a three-layer wax-printed structure, with its core design comprising three asymmetric functional zones (Figure 1A). The top layer serves as a distribution layer, synchronously delivering washing buffer to four detection areas (1 blank control zone and 3 sample detection zones) via wax-printed microchannels to avoid manual pipetting errors. The middle layer acts as the detection layer, immobilizing 5′-amino-modified ctDNA capture probes via chitosan-glutaraldehyde cross-linking, ensuring sandwich-specific hybridization of capture probes-ctDNA-MnB_2_-labeled signal probes. The bottom layer collects unbound probes and waste fluids to prevent signal interference. After target ctDNA binds the capture probes and MnB_2_-labeled signal probes to form a sandwich structure, MnB_2_ catalyzes 3,3′,5,5′-Tetramethylbenzidine (TMB) color development. A smartphone extracts ΔRGB values through the ctDNA Detection APP and outputs concentration based on a standard curve (Figure 1B).

To meet precision and applicability needs for detecting multiple target analytes in complex matrices, researchers have developed a nanozyme colorimetric sensing system based on the activities of phosphatase, peroxidase and oxidase enzymes. In related studies, Jiang et al. developed a colorimetric sensing system based on bimetallic Mn/Fe covalent organic polymer nanozymes (Mn/Fe COP) (Shi YH. et al., 2024), demonstrating high sensitivity for detecting α-glucosidase (α-Glu) and diabetes-related inhibitors (Jiang et al., 2025). It also exhibits superior substrate affinity compared to other products, with a broader tolerance range for pH and temperature than natural laccase, providing a stable foundation for subsequent sensing applications. In terms of sensing mechanisms, it first catalyzes the target enzyme, using α-Glu as the detection target, to specifically hydrolyze the substrate 2-O-α-D-glucopyranosyl-L-ascorbic acid (AAG), decomposing it into glucose and ascorbic acid (AsA) (Lv et al., 2024). The next step involves the inhibitory effect of AsA on the nanozyme-catalyzed reaction, where the reducing AsA can reduce the red quinone product catalyzed by Mn/Fe COP, causing the solution to fade. The higher the α-Glu concentration, the more AsA is generated, and the more pronounced the fading (Figure 1B) (Jiang et al., 2025). By extracting the RGB parameters of the solution using the smartphone Color Grab app and using (B + G)/2R as the quantitative signal, a linear correlation coefficient as high as 0.9994 is obtained (Guo et al., 2024a).

In addition to the above sensing systems, there are other colorimetric integrated sensing systems that focus on catalytic mechanisms and single signal devices to meet different biomedical sensing needs. For example, the FeCoZn TAC/SNC nanozyme developed by Wu et al. possesses enzyme-mimetic activity and can catalyze the oxidation of the chromogenic substrate TMB to generate the colored product oxidized TMB (oxTMB). Ascorbic acid can reduce the colored product to cause color fading, and the signal intensity changes with the concentration of AA (Wu et al., 2022). Li et al.’s H-MnO_2_ nanozyme exhibits multi enzyme activity, which can regulate the oxidative color development of TMB (oxTMB) through enzyme cascade reaction to achieve smart phone assisted detection (Li S. et al., 2023). The above sensing systems all use smart phone quantification technology, combined with the catalytic effect of metal nanozymes for the detection of multi scene biomedical scenes.

### 2.2 Test strip-based single-mode sensing

Metal nanozyme sensing platforms based on test strips play a crucial role in the biomedical field, among which lateral flow immunoassay (LFA) test strips are one of the widely used platforms (Wei et al., 2022; Liu Y. et al., 2021). However, traditional colorimetric labels represented by colloidal gold suffer from limited sensitivity and a narrow detection range, making it difficult to meet the detection requirements for low-concentration biomarkers. In recent years, the emergence of metal nanozymes—which integrate the properties of nanomaterials and enzyme-mimetic catalytic activity—has offered a powerful tool for constructing highly sensitive metal nanozyme sensing platforms based on test strips (Renzi et al., 2023; Wang H. et al., 2024; Tang et al., 2025). By catalyzing the chromogenic reaction of the substrate to achieve signal amplification, metal nanozymes significantly improve the analytical performance of test strips and quantify biomarkers.

Cardiac fatty acid binding protein is one of the key indicators for the early diagnosis of acute myocardial infarction, and its rapid and sensitive detection is crucial for timely treatment (Timmis et al., 2023; Savin et al., 2018). Wang et al. developed a core-shell structure Au@CeO_2_ nanozyme, which can be synthesized on a large scale at room temperature in a one-step method, which has good application prospects (Wang H. et al., 2024). Researchers have used it for the detection of cardiac fatty acid-binding protein, an early marker of acute myocardial infarction. After catalytic amplification of the TMB/H_2_O_2_ chromogenic system, the detection limit reached 0.35 ng/mL, which was far lower than the clinical threshold value of this marker. The results were highly consistent with the results of commercial Enzyme-Linked Immunosorbent Assay kits (R^2^ = 0.98), proving the reliability of the nanozyme test strip platform in the rapid diagnosis of critical and severe diseases.

**FIGURE 2:**
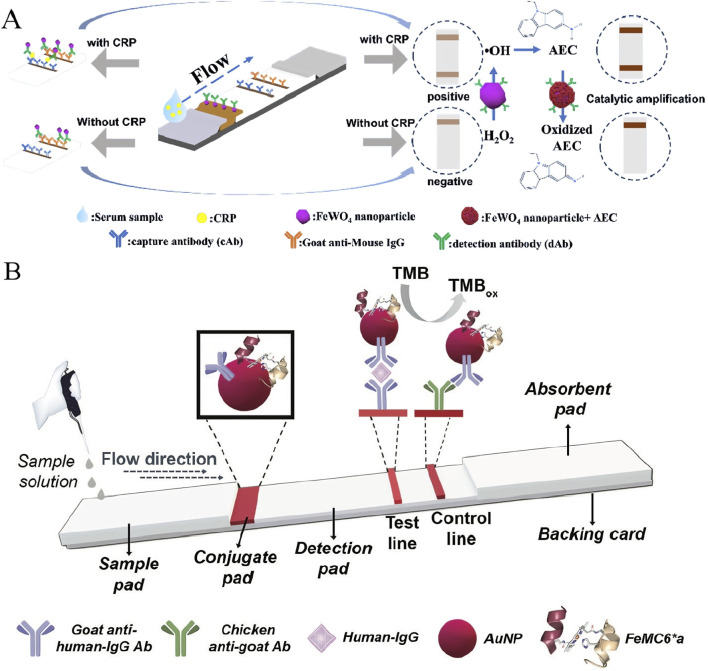
**(A)** Schematic diagram of FeWO_4_ nanoprobe assay concept; **(B)** Schematic representation (not to scale) of FeMC6*a–assisted lateral flow two-step immunoassay for human-IgG detection. Reprinted with permission from Renzi et al. (2023) and Tang et al. (2025).

C-reactive protein (CRP) is a clinically important marker of acute inflammation and cardiovascular disease risk (Sacks et al., 2018; Yang et al., 2020; Huang et al., 2022). Several studies have focused on the highly sensitive detection of nanozyme technology. Tang et al. successfully synthesized an iron-based bimetallic nanomaterial, FeWO_4_ (Tang et al., 2025). The material is prepared by a one-pot hydrothermal method, which has the advantages of excellent peroxidase-like activity, high stability and low cost, and is easy to prepare. During the detection, the sample solution is dropped onto the strip, and the CRP in the solution is first captured by the FeWO_4_ probe and then by the T-line (test line) capture probe. Due to the accumulation of FeWO_4_ probes, gray lines appear on the T line and C line (control line). If CRP is not present, only the C-line is gray. Subsequently, the test strips were immersed in a solution containing hydrogen peroxide and 3-amino-9-ethylcarbazole (AEC), and in the presence of hydrogen peroxide, the AEC was continuously oxidized to brownish-red oxAEC, and the signal of the C and T lines was further enhanced, thus achieving signal amplification (Figure 2A). After catalytic amplification, the detection limit was reduced from 600 ng/mL of traditional colloidal gold test strips to 19.38 ng/mL, and the sensitivity was increased by about 30 times. In addition, the method shows good stability and reproducibility, and has great application prospects in inflammatory diseases. Panferov et al. synthesized a trimetallic nanozyme (Au@Ag-Pt NPs) by an electrosubstitution reaction (Panferov et al., 2021). This method efficiently and uniformly deposits Pt atoms on the surface of Au@Ag core-shell nanoparticles, which greatly improves the utilization rate of precious metal atoms and saves costs. When detecting CRPs, Au@Ag-Pt nanozymes in the immune complex catalyze hydrogen peroxide to oxidize the 3,3′-diaminobenzidine substrate, producing a dark precipitate that significantly enhances the colorimetric signal (Yang et al., 2019).

Human immunoglobulin G is the core of humoral immunity, and its detection is widely used in the diagnosis of infectious diseases, immune assessment, and antibody drug monitoring (Ivanov and Semenova, 2021). Renzi et al. fixed the artificial microperoxidase FeMC6*a on gold nanoparticles to construct a bifunctional probe (Renzi et al., 2023). FeMC6*a is a peptide-porphyrin complex with a well-defined structure and much smaller size than natural horseradish peroxidase. Its small size allows for more active sites to be loaded on gold particles per unit area, resulting in a stronger amplification signal when catalyzing TMB color development. Experiments showed that the FeMC6*a-based test strip increased the detection limit of human IgG from 36.4 ng/mL in the first step (gold particle color development alone) to 8.2 ng/mL after catalytic amplification, which outperformed the control group using natural horseradish peroxidase (Figure 2B). This marks the huge advantages of cleverly prepared artificial enzymes in terms of stability and active site density, opening up a new path for the construction of next-generation test strip sensing platforms.

Single-mode nanozyme sensors currently dominate the nanozyme market, featuring relatively mature technology suitable for various medical applications such as serum testing. Their preparation process is simple and cost-effective, enabling scalable production and convenient portable operation (Tong et al., 2023; Hong et al., 2023; Zhu et al., 2024). However, single-mode nanozyme sensors still face limitations in quantitative accuracy and detection capabilities, as well as issues related to catalytic and reaction stability. For instance, MnB_2_ nanozymes and FeWO_4_ nanozymes struggle to meet the precise quantitative demands for low-abundance biomarkers, such as early-stage tumor ctDNA and trace inflammatory cytokines (Ya et al., 2024; Tang et al., 2025). Meanwhile, most nanozyme systems rely on TMB substrates, whose hydrophobicity and low-temperature coagulation properties can compromise detection stability. Therefore, certain limitations of single-mode nanozyme sensors still require further research and resolution.

## 3 Dual-mode metal nanozyme sensors

**FIGURE 3:**
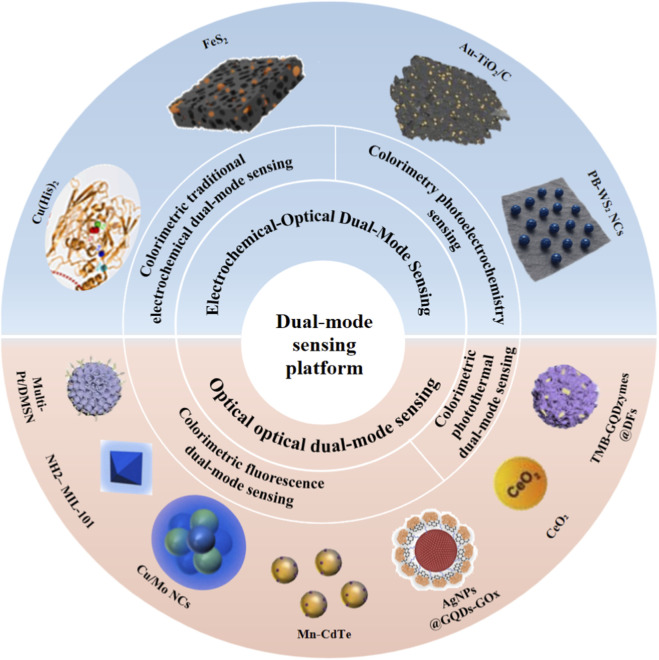
Schematic of dual-mode sensing platform: electrochemical-optical dual-mode sensing through colorimetric traditional electrochemical dual-mode sensing (Reproduced with permission (Madhuvilakku et al., 2025). Copyright 2025, Elsevier. Reproduced with permission (Fu et al., 2023). Copyright 2023, Elsevier) and Colorimetry photoelectrochemistry sensing (Reproduced with permission (Shi et al., 2025). Copyright 2025, American Chemical Society. Reproduced with permission (Chen et al., 2025). Copyright 2025, Elsevier.) as well as Optical dual-mode sensing through Colorimetric fluorescence dual-mode sensing (Reproduced with permission (Hai et al., 2021). Copyright 2025, Elsevier. Reproduced with permission (Qiang et al., 2025). Copyright 2025, Elsevier. Reproduced with permission (Hu et al., 2023). Copyright 2023, Elsevier. Reproduced with permission (Chu et al., 2022). Copyright 2022, Elsevier. Reproduced with permission (Xue et al., 2025). Copyright 2025, Springer-Verlag GmbH Austria, part of Springer Nature) and Colorimetric photothermal dual-mode sensing (Reproduced with permission (He et al., 2023). Copyright 2023, Springer-Verlag GmbH Germany, part of Springer Nature. Reproduced with permission (Zhang et al., 2023). Copyright 2023, Elsevier).

Single-mode metal nanozyme sensors focus on analyzing a single signal, while dual-mode sensors developed from this foundation can integrate and analyze two distinct signals. By leveraging synergistic validation from dual signals, these sensors enhance detection accuracy and interference resistance, while also expanding detection ranges through complementary signals. Common dual-mode signal combinations include “optical-optical dual-signal sensing” and “chemical-optical dual-signal sensing”. This section will elaborate on the latest research concerning these two combinations. Additionally, this section provides a clear comparative overview of the latest research on dual-mode metal nanozyme sensors in the form of pie charts and tables, with a detailed analysis of their respective advantages and disadvantages (Figure 3; Table 2).

**TABLE 2:** Comparison of dual-mode metal nanozyme sensors.

Type		Nanozyme	Simulated enzyme activity	Detection method	Detection target	Advantage	Disadvantage	Ref.
Optical-Optical Dual-Mode Sensing	Colorimetric photothermal dual-mode sensing	CeO_2_	Oxidase-like activity	1) Colorimetry 2) Photothermal	1) AchE 2) Organophosphorus compounds	1) No H_2_O_2_ interference 2) Suitable for real-time detection scene portable detection	Laser irradiation conditions need to be strictly controlled	He et al. (2023)
		TMB-GQDzymes@DFs	Peroxidase-like activity	1) Colorimetry 2) Photothermal	Tumor-derived exosomes	Suitable for real-time detection scene portable detection	Assembly of DNA flowers by rolling circle amplification	Zhang et al. (2023)
	Colorimetric fluorescence dual-mode sensing	AgNPs@GQDs-GOx	Peroxidase-like activity	1) Colorimetry 2) Fluorescence	Glucose	Can activate on/off signal (high signal-to-noise ratio)	GOx load efficiency needs to be improved	Hai et al. (2021)
		Cu/Mo NCs	Peroxidase-like activity	1) Colorimetry 2) Ratiometric fluorescence	Butyrylcholinesterase	Anti interference of ratiometric fluorescence self calibration	Complex assembly process of double nanozyme	Qiang et al. (2025)
		Mn-CdTe	Peroxidase-like activity	1) Colorimetry 2) Fluorescence	Hg^2+^	1) Wide pH range 2) Fluorescence quenching signal is stable	Slightly responsive to other metal ions	Hu et al. (2023)
		NH_2_-MIL-101	Peroxidase-like activity	1) Colorimetry 2) Ratiometric fluorescence	Alendronate sodium	1) Ratio signal anti matrix interference 2) Low detection limit	Store away from light	Chu et al. (2022)
		PtDMSN	Peroxidase-like activity	1) Colorimetry 2) Fluorescence imaging	HER2-positive breast cancer cells	Strong specificity for tumor cells	High cost of platinum based nanozymes	Xue et al. (2025)
Electrochemical-Optical Dual-Mode Sensing	Colorimetric traditional electrochemical dual-mode sensing	Cu(His)_2_	1) Oxidase-like activity 2) Laccase-like activity	1) Colorimetry 2) Electrochemistry	Human neuroblastoma shsy-5y cells	1) Real time monitoring of cells can be achieved 2) Strong anti-interference ability against AA/UA	1) The electrode modification process is complex 2) Dependent on enzyme specificity	Madhuvilakku et al. (2025)
		FeS_2_	3) Oxidase-like activity	1) Colorimetry 2) Electrochemistry	Dopamine	1) Excellent conductivity 2) The detection of serum samples is stable	Selectivity depends on enzyme specificity	Fu et al. (2023)
	Colorimetry photoelectrochemistry sensing	Au-TiO_2_/C	Peroxidase-like activity	1) Colorimetry 2) Photoelectrochemistry	Cholesterol	1) Au TiO_2_ synergistically enhances catalytic activity 2) Good stability in serum sample detection	Relying on cholesterol oxidase cascade to generate H_2_O_2_	Shi et al. (2025)
		PB-WS_2_ NCs	Peroxidase-like activity	1) Colorimetry 2) Photoelectrochemistry	1) Cholesterol 2) H_2_O_2_ 3) CYFRA21-1	Strong adaptability of multi marker detection	Photoexcitation conditions need to be strictly controlled	Chen et al. (2025)

### 3.1 Optical-optical dual-mode sensing

Optical-optical dual-mode sensing, centered on the catalytic activity of nanozymes, this technology targets the detection of biomarkers such as acetylcholinesterase (AChE) and glucose. On the one hand, it utilizes colorimetric signals to achieve rapid visual qualitative analysis (*e.g.*, the blue/colorless change induced by TMB oxidation, and the fading of the brownish-yellow color caused by AgNPs degradation). On the other hand, it accomplishes accurate quantitative analysis through photothermal signals (the near-infrared photothermal conversion of oxTMB) or fluorescence signals (the quenching/recovery of GQDs). The cross-calibration of the dual optical signals can directly avoid optical interferences such as the inherent color of samples and ambient light (Zhou et al., 2020; Lu et al., 2021). Its core design logic involves leveraging the specific catalytic reactions of nanozymes to generate quantifiable dual signals, ultimately achieving rapid and precise quantification of target molecules (Zhang et al., 2023; Hai et al., 2021).

**FIGURE 4:**
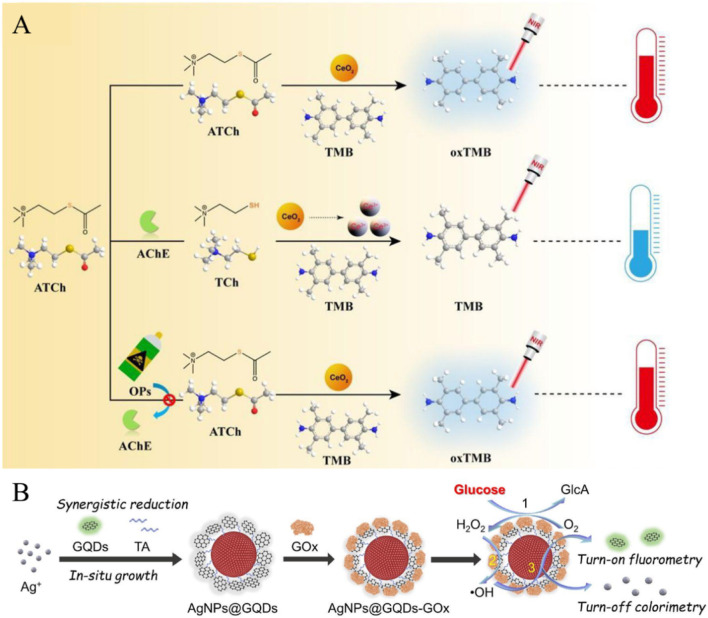
**(A)** CeO_2_ nanozyme colorimetric-photothermal dual-Mode sensing mechanism. **(B)** Catalysis and synergistic therapy mechanism of AgNPs@GQDs-GOx. Reprinted with permission from Hai et al. (2021) and Chu et al. (2022).

The existing liver injury related AChE detection equipment has strong dependence and low reliability. Therefore, He et al. developed a colorimetric photothermal dual-mode sensing system with CeO_2_ nanozyme as the core, which solved the difficulties of traditional detection operations and weak anti-interference (He et al., 2023). By precisely regulating the catalytic and sensing mechanisms, a clear quantitative relationship is established between AChE activity and signal changes: the higher the AChE activity, the more TCh is generated, and its inhibitory effect on CeO_2_ catalysis becomes more significant. This leads to a reduction in oxTMB production via CeO_2_ oxidation, a decrease in photothermal conversion efficiency, and a notable decline in the system’s temperature rise following laser irradiation. Specifically, the absorbance at 652 nm wavelength (blue concentration displayed by oxTMB) decreased, followed by a significant decrease in system temperature increase after 808 nm laser irradiation. The mutual verification of two signals significantly improves the detection accuracy (Figure 4A) (He et al., 2023). Similarly, Zhang et al. developed TMB-GQDzymes@DFs. The system recognizes tumor exosomes through dual aptamers and utilizes GQDzymes to catalyze TMB to generate oxTMB for colorimetric photothermal dual-mode detection (Zhang et al., 2023).

In order to meet the integrated needs of blood glucose detection and tumor diagnosis and treatment, Hai et al. constructed AgNPs@GQDs-GOx. The colorimetric fluorescence dual-mode sensing platform with composite materials as the core has successfully achieved synergistic integration of detection and treatment functions (Hai et al., 2021). This system adopts an efficient cascade catalytic reaction mode, and its catalytic process has been verified by ESR, showing excellent substrate conversion efficiency. Specifically, glucose oxidase (GOx) first recognizes and catalyzes the oxidation of glucose to produce gluconic acid and H_2_O_2_ (Jiang SS. et al., 2019). Graphene quantum dots (GQDs) exhibit highly efficient peroxidase like activity, rapidly catalyzing the decomposition of H_2_O_2_ to produce highly oxidizing ·OH (Jiang SS. et al., 2019; Liu et al., 2017). ·OH further reacts with AgNPs in the composite material, oxidizing AgNPs to Ag^+^, leading to the degradation of AgNPs (Fu et al., 2018). During the sensing process, the dual signals exhibit regular changes with the variation of glucose concentration: the characteristic absorption peak of AgNPs at 403 nm gradually weakens as AgNPs degrade, which serves as the colorimetric signal. Meanwhile, the fluorescence quenching effect of AgNPs on GQDs is relieved via the resonance energy transfer (FRET) mechanism, leading to a gradual recovery of the blue fluorescence of GQDs at 450 nm, which acts as the fluorescence signal (Hai et al., 2021; Yu et al., 2017; Wang et al., 2014). Both signals show a good linear relationship with glucose concentration and can accurately quantify blood glucose levels (Figure 4B) (Yu et al., 2024b). In other similar systems, Abd AlGhafar et al. constructed Fe@GABA CDs nanozyme to achieve chemiluminescence colorimetric dual-mode detection of anticancer drug raloxifene (Abd-AlGhafar et al., 2026). Qiang et al. constructed a ratio fluorescence colorimetric platform to detect BChE enzyme by synergistically combining Cu/Mo NCs with CoFe LDH (Qiang et al., 2025), and showed excellent detection performance.

### 3.2 Electrochemical-optical dual-mode sensing

Nanozymes have become a core research direction in the field of biosensing due to their significant advantages such as high stability and low cost. In recent years, significant breakthroughs have been made in related research: precise detection of dopamine, cholesterol, H_2_O_2_, lung cancer markers, and CYFRA21-1 has been successfully achieved through a dual sensing mode of electrochemistry and optics. These breakthroughs have effectively solved the limitations of traditional detection methods and provided innovative tools for the diagnosis and treatment of nervous system diseases, infectious diseases, metabolic diseases and lung cancer. These advances further highlight the enormous potential of nanozymes in biomedical research (Madhuvilakku et al., 2025; Shi et al., 2025; Chen et al., 2025).

The PolyCuHis sensor developed by Madhuvilakku et al. demonstrates significant practical value in real-time neurotransmitter monitoring and clinical diagnostics (Madhuvilakku et al., 2025). The most significant advantages of this sensor are: the linear range for electrochemical detection of dopamine is 10 nM–100 μM, the detection limit is as low as 2.8 nM, and the peak current is 2.15 times that of the bare electrode. Combining electrochemical and colorimetric dual-mode detection, the detection limit of colorimetric method is 0.204 μg/mL, and the detection limit of smartphone on-site detection is 1.6 μg/mL. The recovery rate of artificial cerebrospinal fluid spiked is 98.43%–105.37%. Its advantages are fully validated by experimental evidence, including ultra-low detection limits, dual-mode signal output, high anti-interference capability, long-term stability, and applications at the cellular level. The core mechanism stems from the synergistic interaction between Cu^2+^ and histidine, which not only mimics the catalytic activity of natural enzymes but also overcomes their instability and cost limitations (Tian et al., 2024; Wang et al., 2025c; Zhong et al., 2023; Sha et al., 2023).

**FIGURE 5:**
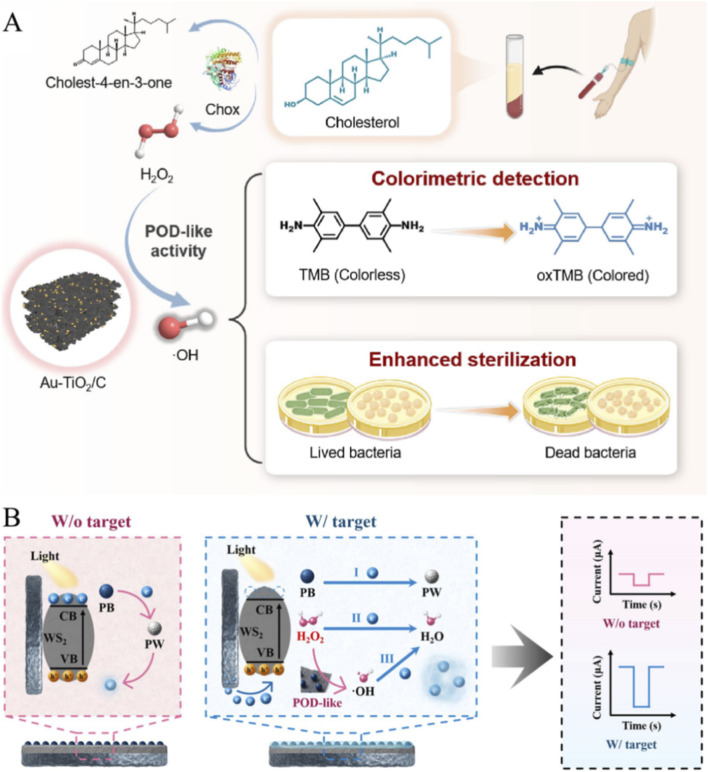
**(A)** Antibacterial properties of cholesterol sensor and Au-TiO_2_/C nanozyme; **(B)** Schematic diagram of Prussian blue-WS_2_ nanozyme detection Reprinted with permission from Shi et al. (2025) and Chen et al. (2025).

Shi et al.'s research has demonstrated the superiorities of Au-TiO_2_/C nanozymes, including high catalytic activity, low H_2_O_2_ dependence, high detection sensitivity, and broad-spectrum antibacterial properties. These outstanding properties provide solid experimental support for the application of Au-TiO_2_/C nanozymes in biosensing and antibacterial therapy (Yuan et al., 2017; Yuan et al., 2018; Yu et al., 2021). The Au-TiO_2_/C nanozyme enhances catalytic performance through electrochemical interfacial electron modulation, combining optical colorimetric detection with fluorescence verification to achieve dual functions of highly sensitive cholesterol detection and efficient bacterial killing (Figure 5A) (Shi et al., 2025). Under the condition of 1 mM H_2_O_2_, the viable cell count of the Au-TiO_2_/C treatment group decreased by 2 orders of magnitude compared to the TiO_2_/C group, and direct morphological evidence of bacterial cell wall rupture and content leakage was observed by scanning electron microscopy. This result directly proves that Au nanoparticle modification enhances peroxidase like activity and significantly improves the antibacterial efficacy of nanozymes. It has important potential in the diagnosis and treatment of infectious diseases and metabolic diseases. Chen et al. developed an electrochemical optical mode bioanalysis platform based on Prussian blue tungsten disulfide nanocomposites (PB-WS_2_ NCs) (Figure 5B) (Chen et al., 2025). Used for colorimetric detection of cholesterol, photoelectrochemical detection of hydrogen peroxide (H_2_O_2_), and lung cancer biomarker cytokeratin 19 fragment (CYFRA21-1), it can be used for comprehensive research on complex diseases such as lung cancer (Guo et al., 2024b). In recent years, there have also been many other studies on electrochemical optical dual-mode sensing (Li H. et al., 2023; Qi et al., 2025; Wang et al., 2025d; Wang S. M. et al., 2024).

Nanozymes are a hot topic in biosensing. Various nanozymes such as transition metal sulfide porous carbon and copper coordination thin films have been developed, which can detect biomarkers such as dopamine and cholesterol. Dual mode sensing is often used to improve accuracy, with advantages such as low detection limit and wide linear range. They are suitable for complex samples and integrate convenient functions, providing support for precise biomedical detection (Luo et al., 2022).

Dual-mode nanozyme sensors enable complementary dual-signal detection, such as verifying results through both absorbance and temperature measurements (Zhang et al., 2023). Compared to single-mode sensors, dual-mode sensors involve a more complex synthesis process. For instance, the preparation of quantum GQDs requires high-temperature hydrothermal reactions, and most nanozyme synthesis processes demand extended reaction times, making large-scale production challenging (Hai et al., 2021).

## 4 Multi-mode metal nanozyme sensors

Metal nanozymes, serving as highly efficient substitutes for natural enzymes, combine the characteristics of nanomaterials with enzymatic catalytic activity. In recent years, they have demonstrated significant application potential in sensing and analytical fields (Jana et al., 2021). Compared to traditional single-mode sensors, multi-mode metal nanozyme sensors enhance analytical accuracy, sensitivity, and applicability by integrating multiple signals or mechanisms, showcasing unique advantages in biomedical applications (Sips et al., 2025; Liang et al., 2023).

**FIGURE 6:**
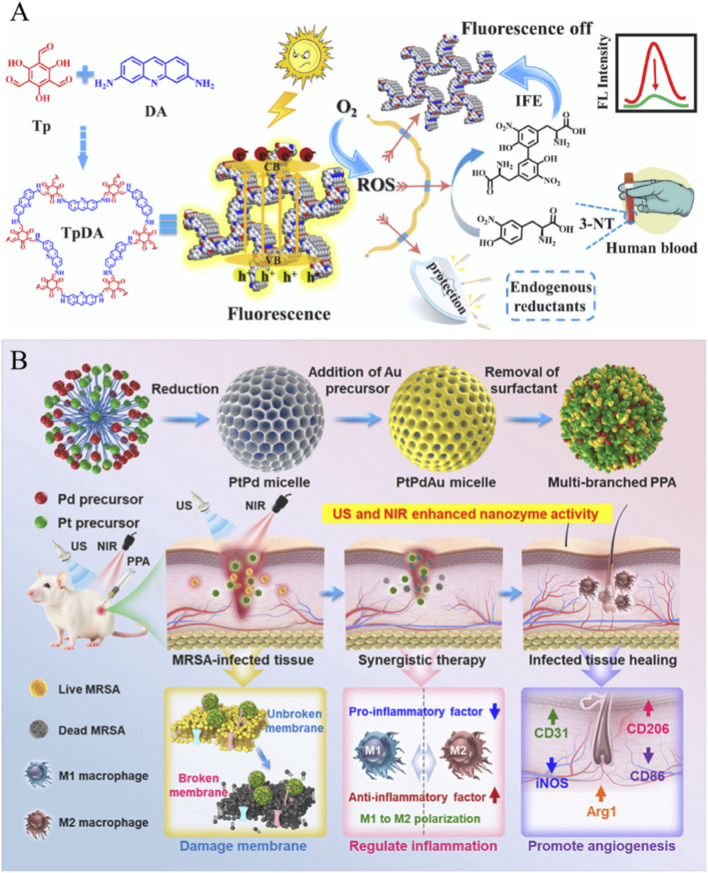
**(A)** Construction of the multifunctional TpDA COF nanozyme for label-free 3-NT detection; **(B)** Schematic diagram of the synthesis of multi-branched PPA alloy nanozyme and the therapeutic processes of deep-seated MRSA infections. Reprinted with permission from Sips et al. (2025) and Liang et al. (2023).

3-Nitrotyrosine is regarded as an important disease marker for tissue and cell damage, and its increased concentration is closely related to various diseases such as atherosclerosis, asthma, myocarditis, meningitis, Parkinson’s disease, etc., (Zhu et al., 2021; Geng et al., 2022). To detect the content of 3-nitrotyrosine in human serum, Liang’s team developed the TpDA COF nanozyme, which successfully integrates three key functions: self-reporting, self-calibration, and photo-response activation. This material inherently possesses fluorescence properties while activating its oxidase activity under light exposure to catalyze the production of reactive oxygen species (ROS) from dissolved oxygen (Song et al., 2010; Cao et al., 2020). When the target analyte 3-nitrotyrosine (3-NT) is present, the intrinsic fluorescence of TpDA is significantly quenched through an intrinsic fluorescence enhancement effect between TpDA and 3-NT’s oxidation products, enabling “switch-type” detection without additional signal probes (Li et al., 2020). Moreover, the excess ROS effectively counteracts interference from endogenous reducing agents (Figure 6A) (Liang et al., 2023). This design circumvents three major challenges in conventional colorimetric methods—H_2_O_2_ instability, O_2_ background interference, and endogenous reductant interference—achieving highly selective and sensitive detection of the disease marker 3-NT in human serum with a detection limit as low as 0.011 μM.

In order to detect and eliminate methicillin-resistant *Staphylococcus aureus* and its biofilm, Wang et al. developed Pt-Pd-Au trimetallic alloy nanozyme (PPA), which showed excellent photothermal conversion efficiency (52.21%) and dual mimic enzyme activities of oxidase and peroxidase through its branched chain structure and electron synergy (Figure 6B) (Shi et al., 2021). PPA can synchronously respond to near-infrared light and ultrasonic external field stimulation, converting photon energy and mechanical waves into a significant thermal effect (ΔT > 20 °C) and enhanced catalytic reactions, respectively. It can also sense the weakly acidic pH and endogenous H_2_O_2_ at the infection site, activate its dual enzyme activity, and explosively generate ROS as the primary response signal. This ROS acts on biological targets together with the aforementioned chemical and physical signals, ultimately causing damage to the bacterial membrane structure. The disintegration of the biofilm and the sharp decrease in pathogen survival (which can be as low as 11.05%) are used as biological signal outputs (Sips et al., 2025). Integrated transcriptomic and proteomic analyses reveal that PPA exerts its effects through multiple mechanisms including regulation of oxidative stress pathways, quorum sensing systems, and metabolic networks (Dasari et al., 2022). This integrated “sensor-therapeutic-regulatory” platform provides innovative solutions for managing deep tissue infections.

In addition to methicillin-resistant *S. aureus*, infections caused by *Streptococcus pneumoniae* and *Klebsiella pneumoniae* have become significant contributors to the rising incidence of community-acquired pneumonia. Traditional detection methods suffer from multiple limitations, including time-consuming procedures, complex operations, and strong condition dependency. To address these challenges, Lin et al. developed a ligand-driven, ring-shaped, epitaxial CuS-Au heterostructure with trifunctional plasmonic nanoenzymes (Lin et al., 2025). The sensor core leverages LFA to achieve biomedical detection through three signal modalities: SERS, catalytic colorimetry, and photothermal sensing. It enables simultaneous rapid visual screening and ultrasensitive quantitative detection of *Streptococcus* pneumoniae and *K. pneumoniae*, meeting the point-of-care testing demands of primary healthcare settings and resource-limited environments.

In summary, the multi-mode metal nanozyme sensor, through clever structural design, successfully integrates multiple sensing functions into one, and plays an important role in many fields such as precision medicine, intelligent and biomedical applications.

## 5 Conclusion and prospective

In summary, metal nanozyme sensors, with their core characteristics of “high sensitivity, fast response, low cost, and easy integration,” have pioneered an innovative technological pathway for efficient sensing signal-assisted therapy in the biomedical field. Through their controllable dimensions, easily modifiable surfaces, and excellent biocompatibility, these materials play a significant role in various disease prevention and treatment applications. This review categorizes metal nanozyme sensors with different sensing modes into three major types: single-mode, dual-mode, and multi-mode. At the conclusion of this review, a systematic comparison of the advantages, disadvantages, and application environments of these three categories of metal nanozyme sensors is presented in tabular form (Table 3). It systematically reviews their latest research and applications in biomedicine, providing a crucial reference for future studies on these diverse sensing modes.

**TABLE 3:** Comparative summary of three different nanozyme sensors.

Sensing mode		Advantage	Disadvantage	Application	Ref.
Single mode sensing	Device-Integrated Single-Mode Sensing	1) Portable and efficient 2) Simple operation 3) Short detection cycle 4) High sensitivity 5) Strong stability 6) Low preparation cost, suitable for large-scale production	1) Strict storage conditions 2) Limited detection range 3) Reliance on complex sample processing	1) Cancer diagnosis and treatment-related testing 2) Metabolic disease screening 3) neurological disease diagnostic support 4) Clinical enzyme activity testing	Yuan et al. (2022), Wu et al. (2022), Jiang et al. (2025), Zhou et al. (2023), Liu et al. (2026), Li et al. (2023a), Lian et al. (2024), Ya et al. (2024)
	Test Strip-Based Single-Mode Sensing	1) High sensitivity 2) Low detection limit 3) Strong specificity 4) Convenient and rapid operation 5) High stability 6) Low cost, suitable for large-scale production 7) Visual signal detection	1) Susceptible to environmental factors 2) High preparation requirements for nanocatalysts 3) Limited detection range	1) Acute inflammation/infection 2) Acute myocardial infarction 3) Immune-related diseases 4) Chronic inflammation-related diseases	Panferov et al. (2021), Renzi et al. (2023), Tang et al. (2025); Wang H. et al. (2024)
Dual-mode Metal Nanozyme Sensors	Optical-Optical Dual-Mode Sensing	1) Dual signal verification, high accuracy 2) High sensitivity 3) High stability 4) Multifunctional integration	1) Risk of signal cross-interference 2) High environmental sensitivity 3) Significant variation in nanozyme preparation and cost 4) Limitations in complex sample adaptation	1) Tumor-related diseases 2) Metabolic and endocrine disorders 3) Liver and kidney function-related diseases 4) Neurological and inflammatory conditions 5) Drug toxicity and monitoring	Zhang et al. (2023), Hai et al. (2021), Hu et al. (2023), Chu et al. (2022), Qi et al. (2025), Qiang et al. (2025)
	Electrochemical-Optical Dual-Mode Sensing	1) Signal complementarity, high accuracy and sensitivity 2) Flexible operation, wide adaptability to scenarios 3) Strong stability and anti-interference capability 4) Multi-functional integration	1) Nanozyme preparation demands high precision 2) High environmental sensitivity	1) Neurological disorders 2) Metabolic and cardiovascular diseases 3) Cancer-related diseases	Madhuvilakku et al. (2025), Fu et al. (2023), Shi et al. (2025), Chen et al. (2025)
Multi-Mode Metal Nanozyme Sensors	​	1) Synergistic enhancement 2) Dual excellence in sensitivity and 3) Interference resistance 4) High functional integration	1) Reliance on external stimuli and specialized equipment 2) Complex nanozyme preparation process 3) Further validation required for clinical translation	1) Infectious diseases 2) Inflammation and tissue injury-related diseases	Sips et al. (2025), Liang et al. (2023)

Metal nanozymes offer significant advantages for sensing applications due to their small size, controllable structure, and ease of functional integration. Single-mode sensors stand out for their operational convenience. Among these, metal nanozyme sensors utilizing smartphones as core sensing platforms enable instant quantitative analysis through digital signal conversion, greatly expanding applications in point-of-care diagnostics and home health monitoring. Immunochromatographic sensors based on test strips excel in rapid visualization. Leveraging the catalytic signal amplification effect of metal nanozymes, they can complete qualitative and quantitative analysis within minutes, meeting the efficiency demands of primary healthcare and emergency testing. Additionally, dual-mode sensors overcome the limitations of single modes through complementary signals, achieving superior functional integration. Optical-optical dual-mode sensing integrates two optical signals, effectively mitigating background interference from biological samples and enhancing detection accuracy of target molecules in complex matrices. Optical-electrochemical dual-mode sensing combines the intuitiveness of optical signals with the high sensitivity of electrochemical signals, providing more comprehensive diagnostic evidence for early disease detection. Furthermore, multi-mode sensors, as an advanced development form, not only inherit the core advantages of single and dual modes but also significantly enhance the accuracy and fault tolerance of complex disease diagnosis through multidimensional signal cross-validation and complementarity. In summary, metal nanozyme sensors leverage the flexible adaptability of diverse sensing modes to meet simple application needs such as point-of-care testing and primary screening. They also enable precise quantitative analysis and multidimensional assessment of target molecules in complex biological matrices through the integration of multiple signals. This versatility accommodates diverse biomedical applications ranging from routine screening to dynamic monitoring of complex diseases.

Currently, multiple metal nanozymes have demonstrated outstanding efficacy and safety in animal models or complex biological samples across scenarios such as tumor therapy, inflammatory disease intervention, and clinical metabolite monitoring, confirming their clinical applicability in disease diagnosis and treatment. Regarding the trade-off between catalytic activity and biocompatibility, synergistic optimization of catalytic efficiency and biosafety has been achieved through strategies such as multi-metal synergistic design and surface functionalization. In terms of performance evaluation under physiological conditions, existing research has clarified the regulatory mechanisms of physiological factors like pH and biological matrix components on nanozyme catalytic activity. Furthermore, critical issues such as insufficient stability have been effectively addressed through strategies like dynamic response design.

Despite substantial progress in metal nanozyme sensor research, significant challenges persist. Furthermore, the lack of standardized preparation processes and quality control systems in practice makes it difficult to ensure material performance uniformity during large-scale production. Additionally, the poor compatibility of existing detection systems with routine clinical testing workflows limits their adoption in medical institutions. Metal nanozyme sensors have yet to achieve comprehensive clinical translation and require further advancement. Concurrently, global regulatory frameworks are progressively evolving to establish core metrics for materials, performance, and safety. Synergizing research with industrial production to form a closed-loop technology transfer system can effectively drive process optimization and cost reduction, thereby enhancing applicability in pilot programs for primary healthcare and precision medicine. To accelerate clinical translation, comprehensive solutions must be developed across multiple dimensions. At the processing technology level, precise and controllable nanoscale fabrication techniques should be developed. Surface functionalization modifications should optimize material biocompatibility and targeted recognition capabilities while reducing non-specific binding. Simultaneously, automated production lines and stringent quality audit standards must be established to quantitatively test critical metrics such as material dimensions, catalytic activity, and stability, ensuring batch-to-batch consistency. Furthermore, enhanced collaboration with clinical institutions is essential to optimize sensor operation workflows for clinical testing scenarios, aligning them with routine practices in healthcare settings. This requires clear translation strategies and financial backing, representing the most time-consuming and challenging phase of the entire process.

Metal nano-enzyme sensors hold immense potential in biomedical applications, with future development goals centered on high specificity, multifunctionality, and intelligent capabilities. Deep integration with emerging technologies will further expand their application boundaries. Combining them with wearable devices and flexible electronics enables portable sensor systems for real-time physiological monitoring, providing continuous support for chronic disease management and health alerts. Developing smartphone-assisted detection platforms, such as multilayer microfluidic paper-based analytical devices, can meet the stability requirements for portable testing. In the future, 3D printing technology will enable rapid fabrication of customized microfluidic chip sensors to address personalized medical testing needs. Continuous breakthroughs in artificial intelligence will advance the intelligent analysis of multidimensional sensory signals, enhancing the accuracy and predictive capabilities of disease diagnosis.

In summary, we believe that despite ongoing challenges, the integration of emerging technologies is accelerating innovation in the field of metal nano-enzyme sensors. Significant progress has been made over the past 5 years. Looking ahead, metal nano-enzyme sensors will increasingly replace traditional sensors in diverse applications, exerting a profound impact on biomedicine and health management. We anticipate further exciting breakthroughs in this material in the coming years.
